# Assessment of Sepsis Risk at Admission to the Emergency Department: Clinical Interpretable Prediction Model

**DOI:** 10.3390/diagnostics14050457

**Published:** 2024-02-20

**Authors:** Umran Aygun, Fatma Hilal Yagin, Burak Yagin, Seyma Yasar, Cemil Colak, Ahmet Selim Ozkan, Luca Paolo Ardigò

**Affiliations:** 1 Department of Anesthesiology and Reanimation, Malatya Yesilyurt Hasan Calık State Hospital, Malatya 44929, Turkey; umranaygun92@gmail.com; 2 Department of Biostatistics and Medical Informatics, Faculty of Medicine, Inonu University, Malatya 44280, Turkey; seyma.yasar@inonu.edu.tr (S.Y.); cemil.colak@inonu.edu.tr (C.C.); 3 Department of Anesthesiology and Reanimation, Malatya Turgut Ozal University School of Medicine, Malatya 44210, Turkey; ahmet.ozkan@ozal.edu.tr; 4 Department of Teacher Education, NLA University College, 0166 Oslo, Norway; luca.ardigo@nla.no

**Keywords:** sepsis, machine learning, explainable artificial intelligence, biomarker

## Abstract

This study aims to develop an interpretable prediction model based on explainable artificial intelligence to predict bacterial sepsis and discover important biomarkers. A total of 1572 adult patients, 560 of whom were sepsis positive and 1012 of whom were negative, who were admitted to the emergency department with suspicion of sepsis, were examined. We investigated the performance characteristics of sepsis biomarkers alone and in combination for confirmed sepsis diagnosis using Sepsis-3 criteria. Three different tree-based algorithms—Extreme Gradient Boosting (XGBoost), Light Gradient Boosting Machine (LightGBM), Adaptive Boosting (AdaBoost)—were used for sepsis prediction, and after examining comprehensive performance metrics, descriptions of the optimal model were obtained with the SHAP method. The XGBoost model achieved accuracy of 0.898 (0.868–0.929) and area under the ROC curve (AUC) of 0.940 (0.898–0.980) with a 95% confidence interval. The five biomarkers for predicting sepsis were age, respiratory rate, oxygen saturation, procalcitonin, and positive blood culture. SHAP results revealed that older age, higher respiratory rate, procalcitonin, neutrophil–lymphocyte count ratio, C-reactive protein, plaque, leukocyte particle concentration, as well as lower oxygen saturation, systolic blood pressure, and hemoglobin levels increased the risk of sepsis. As a result, the Explainable Artificial Intelligence (XAI)-based prediction model can guide clinicians in the early diagnosis and treatment of sepsis, providing more effective sepsis management and potentially reducing mortality rates and medical costs.

## 1. Introduction

Sepsis is a complex syndrome characterized by a dysregulated host response to infection, leading to severe and potentially fatal organ dysfunction. The syndrome’s lethality significantly surpasses that of simple infections, highlighting the imperative for swift recognition and intervention. Early stages of sepsis, even with minor organ dysfunction, are associated with an in-hospital mortality rate exceeding 10% [1,2], emphasizing the critical need for prompt and accurate identification of the syndrome [3]. Sepsis is caused by an imbalance between pro-inflammatory and anti-inflammatory mediators, resulting in a systemic inflammatory response syndrome (SIRS) that can impair the function of multiple organs, such as the lungs, kidneys, liver, heart, and brain. The severity of sepsis is classified according to the presence and number of organ failures, as well as the degree of hypotension and lactate elevation [1]. The diagnosis of sepsis is based on clinical criteria, such as fever, tachycardia, tachypnea, and altered mental status, as well as laboratory tests, such as blood cultures, inflammatory markers, and lactate levels [3]. The treatment of sepsis consists of early administration of appropriate antibiotics, fluid resuscitation, vasopressors, and supportive care for organ dysfunction. The timely initiation of these interventions can reduce the mortality and morbidity associated with sepsis [3,4].

Sepsis is a major challenge for healthcare systems in Turkey and worldwide, requiring prompt diagnosis, appropriate antimicrobial therapy, and supportive care to improve outcomes and reduce mortality. According to the World Health Organization (WHO) data, sepsis affects about 50 million people every year and 11 million of them lose their lives. In Turkey, the prevalence of sepsis varies roughly between 0.5% and 1% [5]. Despite the absence of a universally accepted gold standard for diagnosis, various definitions and scoring systems have been devised to aid in the rapid detection and diagnosis of sepsis. The Sequential Organ Failure Assessment (SOFA) score is a widely validated tool for assessing mortality risk and provides clear bedside criteria for identifying sepsis in adults [1]. Organ dysfunction is indicated by an acute change in the total SOFA score, specifically an increase of two or more points consequent to an infection. Typically, patients with no pre-existing organ dysfunction are presumed to have a baseline SOFA score of zero, and a score of two or higher correlates with a mortality risk of about 10% in a general hospital population with suspected infection. The potential for rapid deterioration even in cases of modest dysfunction underscores the urgency of timely and appropriate clinical responses [6].

For patients with suspected infection and the risk of prolonged intensive care unit (ICU) stays or adverse outcomes, the Quick Sequential Organ Failure Assessment (qSOFA) criteria can be used for early bedside evaluation [6]. These criteria include changes in mental status, systolic blood pressure of 100 mm Hg or less, or a respiratory rate of 22/min or more. However, the sensitivity of qSOFA for detecting early-stage sepsis in certain patient populations has been questioned, potentially delaying the initiation of necessary treatments [7]. Consequently, the 2021 Surviving Sepsis Campaign guidelines recommend against the exclusive use of qSOFA as a solitary screening tool for sepsis. In clinical practice, the integration of blood biomarkers may provide additional valuable information for identifying high-risk patients or those progressing toward organ failure, even when presenting with low SOFA scores [4]. From this clinical point, the SOFA scale is a clinical marker for sepsis prognosis [8]. Similarly, the Acute Physiology and Chronic Health Evaluation II (APACHE II) score exhibits efficacy in prognosticating the mortality risk associated with sepsis, with a positive correlation observed between score elevation and increased likelihood of death [9].

Recent advancements in Explainable Artificial Intelligence (XAI) have shown promising results in the early prediction of sepsis, a critical area in healthcare that demands timely and accurate decision-making. XAI models, by offering transparency in their decision processes, enable clinicians to understand and trust the predictions made by AI systems [10]. This is particularly vital in sepsis prediction, where the interpretability of AI models aids in identifying the onset of sepsis, potentially improving patient outcomes. Furthermore, XAI facilitates the identification of key clinical variables and their interactions, enhancing the ability of healthcare professionals to make informed decisions [11]. The integration of XAI in sepsis prediction not only augments the accuracy of diagnoses but also aligns with the ethical need for transparency and accountability in AI applications in healthcare [10]. Recognizing these benefits, the current study focuses on leveraging the capabilities of XAI to develop a more effective and reliable tool for healthcare professionals in the battle against sepsis. In addition, although there are many studies on machine learning-based sepsis classification in the literature, there are very few studies on classification with explainable artificial intelligence and candidate biomarkers [12,13,14,15]. This study would contribute to the literature in this respect. Therefore, the study aims to construct an XAI classification model for predicting the status of sepsis based on the candidate biomarker features.

## 2. Materials and Methods

### 2.1. Data Source

In this study, an open-access dataset of a prospective observational study on adult patients with and without sepsis was used. All patients aged 18 years and older (18–100) who presented to the emergency department with suspected sepsis were included [16]. A total of 1572 adult patients were studied; of these, 560 tested positive for sepsis, while 1012 tested negative. Using the Sepsis-3 criteria, we examined the diagnostic performance characteristics of sepsis biomarkers, both individually and in combination, for confirmed sepsis diagnosis. This study received ethical approval from the Inonu University Non-Interventional Clinical Research Institutional Review Board (decision no: 2023/5215). Informed consent was obtained from all subjects participating in the study.

### 2.2. Outcome Measures

The current study evaluated the presence and absence of sepsis as outcome measurements based on Sepsis-3 criteria. Sepsis was assessed as follows [16].

- Organ dysfunction was identified as an acute change in total SOFA score ≥ 2 points consequent to the infection.
- The baseline SOFA score was assumed to be zero in patients not known to have pre-existing organ dysfunction.
- The confirmed bacterial infection for Sepsis-3 was defined as a clinical infection, identification of relevant bacteria through culture, and a positive blood culture for bacteremia.

### 2.3. Biostatistical Analyses

Analytical (Shapiro–Wilk test) and visual (histogram and probability graphs) techniques were used to assess the quantitative characteristic eligibility for a normal distribution. The interquartile range (IQR) and median were used to describe the quantitative data since they were not normally distributed, and the Mann–Whitney U test was employed to compare the two groups. If a *p*-value was less than 0.05, it was deemed statistically significant. Cohen’s D was used to calculate the effect size. The following thresholds were taken into account while determining the effect size: Cohen proposed that an impact size of d = 0.2 be categorized as “small”, 0.5 as “medium”, and 0.8 as “large” [17]. For qualitative measures, frequency (n) and percentage (%) values were computed, and the chi-square test was used to look at the associations between these features. The statistical analysis was carried out with IBM Corp.’s SPSS 28.0, located in Armonk, NY, USA.

### 2.4. ML Models and Validation

The methodology for assessing the predictive power of machine learning techniques for sepsis is described in this section. The random forest approach was used to approximate the missing values. Within the research, there was an issue of class imbalance in the distribution of sepsis (560 patients with sepsis and 1012 patients without sepsis). The class imbalance problem was resolved by applying the Synthetic Minority Oversampling Technique for Nominal and Continuous (SMOTE-NC). When dealing with real-world data, class imbalance is a prevalent issue that can be characterized as one in which the number of instances in the majority class is much more than the number of cases in the minority class. Balanced data are important because machine learning models might be biased towards the majority class, leading to problems with underfitting or overfitting. SMOTE-NC was applied only to the training set. The second phase was identifying the most important sepsis biomarkers using the ML-based Least Absolute Shrinkage and Selection Operator (LASSO) approach. A popular regularization and feature selection strategy in machine learning and linear regression is called LASSO. Its purpose is to keep regression models from overfitting by choosing a subset of the most pertinent characteristics from a wider range of features. Following preprocessing, the data were split into 80% training and 20% testing, with 10-fold cross-validation (CV) serving as the resampling technique throughout the training phase. This allowed the ML models to be validated. Three models were trained and evaluated using the tree-based AdaBoost, LightGBM, and XGBoost algorithms to find the best model for sepsis prediction. The optimal hyperparameters of each model were determined by Grid Search with 5-fold and 10 repeated k-Fold CV. Accuracy, sensitivity, specificity, F1 score, positive predictive value (PPV), and negative predictive value (NPV) were computed to evaluate and contrast the efficacy of the best ML model in predicting sepsis among various techniques.

### 2.5. Synthetic Minority Over-Sampling Technique (SMOTE)

The “Synthetic Minority Over-sampling Technique”, or SMOTE for short, is a method used in data mining and ML to solve the issue of unbalanced datasets. One class (the minority class) contains substantially fewer examples than another (the majority class) in datasets that are unbalanced. As a result, ML models may perform poorly if they have a bias in favor of the majority class. SMOTE is a resampling method that generates artificial instances of the minority class in an effort to balance the distribution of classes. It functions by creating artificial samples that resemble instances of minority classes that already exist. SMOTE improves the dataset suitability for training ML models by reducing class imbalance and increasing the amount of samples from minority classes [18,19].

### 2.6. Extreme Gradient Boosting (XGBoost)

XGBoost is a powerful and versatile ML algorithm, renowned for its exceptional performance in various predictive modeling tasks. It falls under the category of gradient boosting algorithms, which sequentially combine a set of weak predictive models to create a strong ensemble model. The remarkable success of XGBoost can be attributed to several key innovations. First, it employs a novel regularization term that mitigates overfitting, enhancing model generalization. Furthermore, it optimizes computational efficiency by utilizing a data structure known as a “sparsity-aware block structure” and a technique called “column block compressed sparse column”. These innovations enable XGBoost to efficiently handle large datasets. The algorithm also utilizes a weighted quantile sketch, improving accuracy when selecting splitting points during tree construction. Overall, XGBoost’s combination of regularization, computational efficiency, and accurate splitting point selection has made it a popular choice for a wide range of machine-learning applications [20,21].

### 2.7. Light Gradient Boosting Machine (LightGBM)

LightGBM represents another cutting-edge gradient-boosting algorithm designed for efficient and high-performance ML tasks. It sets itself apart through its unique approach to tree construction, which differs from traditional depth-first or level-wise strategies. LightGBM utilizes a histogram-based approach, in which data are divided into histograms during tree construction, allowing for more efficient computation of gradient and Hessian values. This approach significantly reduces memory usage and speeds up the training process. LightGBM also introduces exclusive features such as “Gradient-based One-Side Sampling” and “Exclusive Feature Bundling”, which further enhance training efficiency and enable the algorithm to tackle high-dimensional data effectively. Its ability to handle large datasets efficiently and its focus on minimizing memory consumption have made LightGBM a popular choice, particularly in real-time and large-scale ML applications [22,23].

### 2.8. Adaptive Boosting (AdaBoost)

AdaBoost is a classical ensemble learning technique that emphasizes adaptive model combinations to improve predictive accuracy. The central idea behind AdaBoost is to iteratively train a series of weak learners and assign them different weights based on their performance. In each iteration, AdaBoost assigns higher weights to the misclassified instances from the previous iteration, effectively forcing the algorithm to focus on the most challenging data points. By giving more weight to the errors, AdaBoost continually adapts and evolves its ensemble of weak learners, ultimately leading to a stronger, more accurate model. The final prediction is a weighted combination of the individual weak learner predictions, with higher-performing weak learners having more influence. AdaBoost has proven effective in boosting the performance of various base classifiers, making it a valuable tool in the ensemble learning toolbox [24,25].

### 2.9. Metrics Used to Evaluate the Performance of ML Models

Accuracy: Accuracy is a classification metric that measures the overall accuracy of a prediction model’s decisions. This criterion is simple and intuitive, but may not be suitable for unbalanced data sets where one class is significantly superior to the other [26].

F1-Score: The F1-Score is a statistic that yields a single number by combining recall (sensitivity) and precision. When working with unbalanced datasets, it is helpful. The model’s recall—its capacity to recognize all pertinent occurrences of the positive class—and precision—its capacity to prevent false positives—are both balanced by the F1-Score [27].

Sensitivity: This metric calculates the proportion of true positive samples that are predicted as positive by the ML model [26].

Specificity: Specificity measures the proportion of actual negative instances that are correctly predicted as negative by the model. It is the ratio of true negatives to the total number of actual negatives [26].

Negative Predictive Value (NPV): NPV measures the proportion of instances predicted as negative that are actually true negatives. It is the ratio of true negatives to the total number of instances predicted as negative [28].

Positive Predictive Value (PPV): PPV is a classification metric that measures the proportion of instances predicted as positive by a model that are actually true positive instances. In other words, PPV assesses the accuracy of the positive predictions made by a model. It is particularly relevant when the cost or consequences of making false positive predictions are significant [28].

Area Under the Receiver Operating Characteristic Curve (AUC): AUC is a metric that evaluates the ability of a classification model to distinguish between classes, particularly in binary classification problems. The ROC curve is a graphical representation of the true positive rate (sensitivity) against the false positive rate (1-specificity) at various threshold settings. AUC calculates the area under this curve, providing a single value that summarizes the model’s ability to discriminate between positive and negative instances. A higher AUC value (closer to 1) indicates better discrimination and performance of the model [26,29].

Brier Score: The Brier Score measures the accuracy of probabilistic predictions made by a model. It is commonly used for assessing the calibration of predicted probabilities in binary or multi-class classification problems. The Brier Score is calculated as the mean squared difference between predicted probabilities and the actual outcomes. It penalizes both overconfidence (assigning high probability to the wrong class) and under confidence (assigning low probability to the correct class). The Brier Score ranges from 0 to 1, with 0 indicating perfect accuracy and 1 indicating the worst possible accuracy. Lower Brier Scores are preferable, indicating better-calibrated probability predictions [26,30].

## 3. Results

In the study, 1572 patients aged between 18 and 100 were examined. Of the patients, 44.3% were female and 55.7% were male. The median haemoglobin (g/L) of female patients was 126 and the median haemoglobin (g/L) of male patients was 135, while statistical tests showed that hemoglobin (g/L) levels were significantly higher in males than in females (*p* < 0.001). Age, systolic blood pressure (mmHg), respiratory rate (breaths/min), oxygen saturation (%), heart rate (beats/min), body temperature (°C), hemoglobin (g/L), leukocyte particle concentration (×10^9^ cells/L), C-reactive protein(mg/L), procalcitonin (ng/mL), neutrophil–lymphocyte count ratio, lactate (mmol/L), intensive care unit, positive blood culture, and systemic inflammatory response syndrome are important biomarkers in sepsis following LASSO.

Table 1 provides a detailed summary of the descriptive statistics and effect size estimations for the sepsis biomarkers found after applying LASSO. The robustness of the feature selection procedure and the potential importance of these biomarkers in the setting of sepsis were reinforced when it was discovered that *p* values for every one of the chosen biomarkers were statistically significant with *p* < 0.05. On examining Table 1, it was seen that the group with sepsis had a considerably greater median age than the group without sepsis. The sepsis group had substantially higher levels of respiratory rate (breaths/min), heart rate (beats/min), body temperature (°C), leukocyte particle concentration (×10^9^ cells/L), C-reactive protein(mg/L), procalcitonin (ng/mL), neutrophil–lymphocyte count ratio, and plaque (mmol/L) (*p* < 0.05). Conversely, the sepsis group had substantially lower levels of hemoglobin (g/L), oxygen saturation (%), and systolic blood pressure (mmHg) (*p* ≤ 0.05). Procalcitonin (ng/mL) had the largest significant effect size (ES: 0.0891) among the chosen biomarkers, according to our study. This implies that procalcitonin (ng/mL) functions as an effective sepsis-positive and sepsis-negative group discriminator and, as such, merits more research as a possible target for therapy or diagnostic biomarker.

In Table 2, the optimum values of the parameters optimized by Grid Search for the three ML models are given.

Three different ML models (AdaBoost, LightGBM, and XGBoost) were created using the important biomarkers of sepsis determined with the help of LASSO, and the prediction performances of these models were compared. Based on the findings of accuracy, F1 score, sensitivity, specificity, PPV, NPV, AUC, and Brier score, all the prediction models performed comparably. Optimum prediction was performed using the XGBoost model, one of the three tree-based ML classifiers. The performances of the models using the original data were lower than the models created after SMOTE-NC, and when the performance measures in the models using the original data were examined, it was observed that the results were biased and inconsistent. After SMOTE-NC, with a 95% confidence range, the XGBoost model produced accuracy of 0.898 (0.868–0.929) and an AUC of 0.940 (0.898–0.980).

Furthermore, the XGBoost model demonstrated exceptionally high specificity of 0.891 (0.837–0.932) and sensitivity of 0.905 (0.854–0.943). A lower false negative (FN) value is associated with a greater sensitivity rating. In comparative biological studies, mistakes including false positives and false negatives are frequent. This finding is significant since one of the primary objectives of this study was to reduce the number of false negatives, or missing sepsis patients (Table 3).

**Figure 1:**
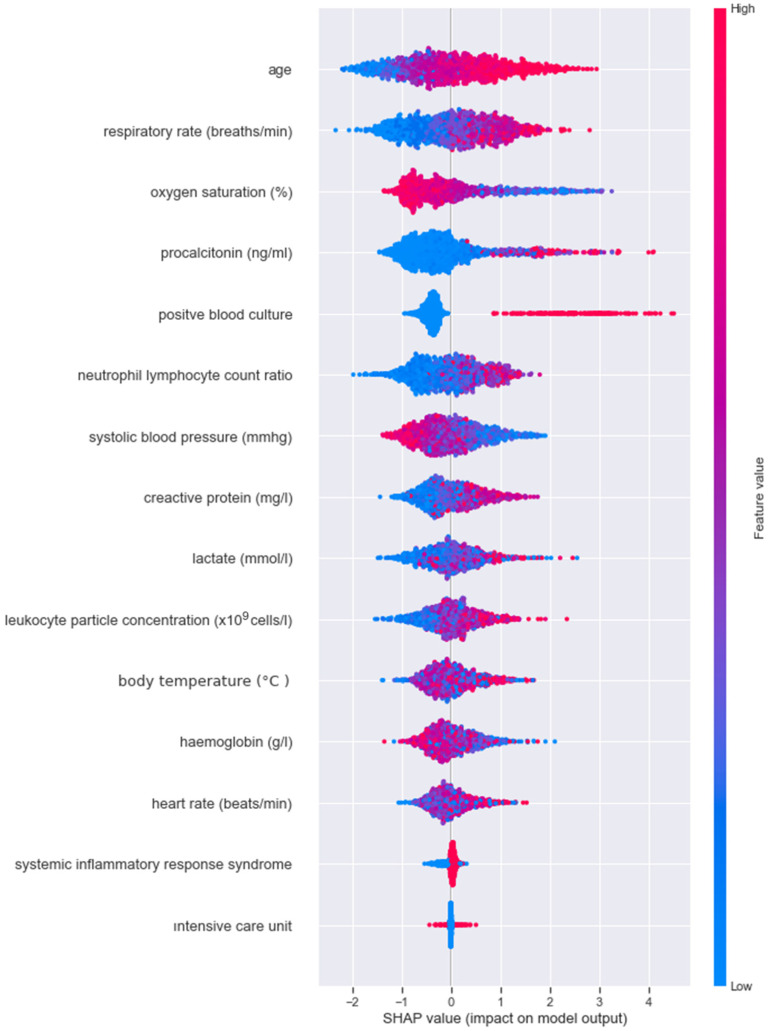
XGBoost model SHAP annotations; The importance of biomarkers is ranked by average (|SHAP value|); the graph’s points are colored based on the normalized values of each patient’s level of biomarker value. The feature value increases as it gets closer to pink and drops as it gets closer to blue. Sepsis is more likely when a feature’s SHAP value is greater. The SHAP plot background for the optimal prediction model is drawn in grey.

**Figure 2:**
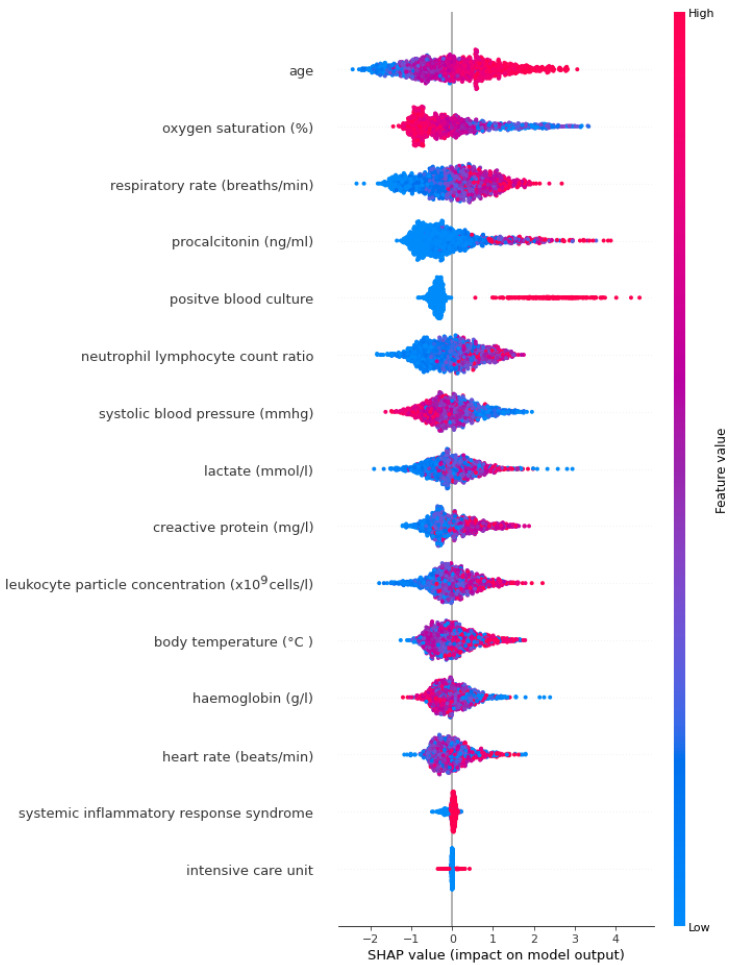
LightGBM model SHAP annotations.

**Figure 3:**
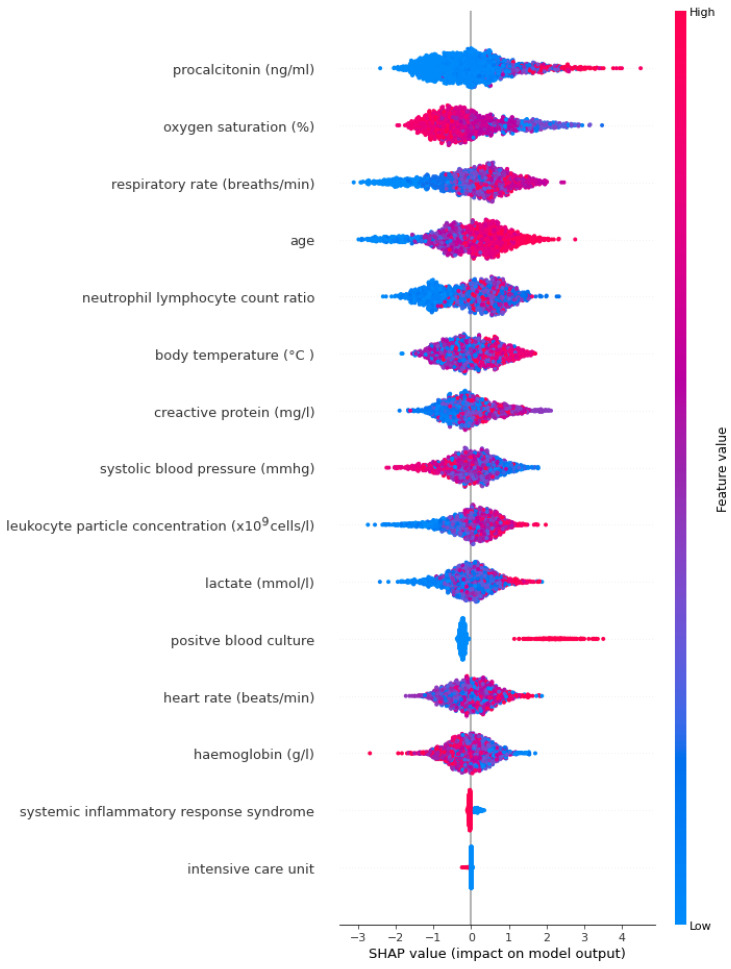
AdaBoost model SHAP annotations.

SHAP annotations were examined to interpret sepsis prediction results of the three tree-based models. With the help of SHAP, we were able to determine the levels of biomarkers important in predicting sepsis, identified by LASSO. The SHAP annotations of the optimal model XGBoost (Figure 1) and the LightGBM (Figure 2) model were more similar compared to the annotations for AdaBoost (Figure 3). According to the explanations of the XGBoost model, age, respiratory rate (breaths/minute), oxygen saturation (%), procalcitonin (ng/mL) and positive blood culture were determined as the five most important biomarkers in the early diagnosis of sepsis. In addition to old age there were higher respiratory rate (breaths/minute), procalcitonin (ng/mL), neutrophil–lymphocyte count ratio, C-reactive protein (mg/L), lactate (mmol/L), leukocyte particle concentration (×10^9^ cells/L) It was determined that body temperature (°C) levels were associated with the risk of sepsis. Additionally, SHAP findings revealed that low oxygen saturation (%), systolic blood pressure (mmHg) and hemoglobin (g/L) levels increased the risk of sepsis (Figure 1).

## 4. Discussion

Sepsis is a critical health condition triggered by an over-activation of the immune system to maintain its normal function, exceeding its capacity to fight widely disseminated infections. This pathophysiological state is characterized by an immune system response to prevent a local infection from having a systemic impact. Sepsis can lead to severe organ dysfunction and life-threatening complications caused by an excessive immune response. This complex process involves important factors that determine the clinical course of sepsis, affecting the ability to control infection and maintain a balanced inflammatory response [31,32]. Economically, sepsis has serious negative impacts due to high treatment costs, prolonged hospitalizations, and rehabilitation processes. An increase in sepsis cases puts financial pressure on health systems, strains hospital resources, and can push intensive care units to their limits. Globally and in Turkey, sepsis is challenged by expenditures and resource allocations that negatively impact economic growth. This emphasizes that sepsis is a significant burden on national economies as well as the health of individuals. In this context, the development of sepsis prevention strategies and effective treatment modalities is critical both to protect the health of individuals and to strengthen economic sustainability [33]. APACHE II and SOFA are two different clinical scales used to assess the severity and prognosis of intensive care unit patients. Both are designed to monitor and guide the treatment of patients in intensive care. The SOFA assesses the function of various organ systems and is used to identify patients with organ failure. The SOFA score is based on six different parameters that assess the function of the respiratory, cardiovascular, hepatic, coagulation, neurological, and renal systems. On the other hand, APACHE II is a scoring system based on the patient’s physiologic status, chronic health status, and age. The APACHE II score is used to predict the patient’s mortality risk and includes many parameters such as the oliguria system, cardiovascular system, neurologic status, and other physiologic measures. Sepsis is among the leading causes of infection-related deaths, such as COVID-19, and scoring systems such as SOFA and APACHE II are used to assess the severity and prognosis of the disease in patients who develop sepsis. Especially in patients with the severe form of COVID-19, these scoring systems can play an important role in directing intensive care resources and determining the treatment plan [34,35,36].

Anticipating sepsis can lower medical expenses and save lives by delaying the onset of multi-organ failure, decreasing admissions to critical care units, and enhancing patient outcomes. Therefore, early prediction of sepsis and initiation of treatment is vital to prevent mortality. Early identification of patients at high risk of sepsis with artificial intelligence (AI) algorithms can significantly improve health outcomes and treatment processes by enabling rapid intervention in intensive care units and halting disease progression. In this study, an artificial intelligence model (XGBoost, LightGBM, AdaBoost) was used to predict bacterial sepsis classified according to SOFA score. XGBoost has the highest performance metrics compared to the other two methods, with accuracy, F1-score, sensitivity, specificity, positive predictive value, negative predictive value, AUC, and Brier score values being 89.8%, 90.1%, 90.5%, 89.1%, 89.6%, 90.1%, 94.0%, and 0.018, respectively. According to the XGBoost model, the five possible biomarkers that can be used to predict sepsis are age, respiratory rate (breath/min), oxygen saturation (%), procalcitonin (ng/mL), and positive blood culture.

Age is an important factor that determines the resistance of individuals to infections. Advanced age is characterized by a series of changes that often lead to an age-related weakening of the immune system. Age-related immunosenescence involves a functional decline in immune cells, which can lead to a reduction in an effective defense mechanism against infections [37]. With advanced age, the increasing incidence of chronic diseases (especially diabetes, cardiovascular disease, and chronic renal failure) can reduce the body’s defense capacity against bacterial infections. This can increase the risk of developing sepsis. With advanced age, the increasing incidence of chronic diseases (especially diabetes, cardiovascular disease, and chronic renal failure) can reduce the body’s defense capacity against bacterial infections. This can increase the risk of developing sepsis [38]. On the other hand, the decline in the function of many organs such as the heart, lungs, and kidneys with increasing age can reduce the effectiveness in fighting infections. Affecting these organs can increase the severity of the sepsis process. Furthermore, in a systematic review of 17 articles, age was reported to be one of the most important predictors among 194 predictors [39].

Sepsis causes widespread inflammation, leading to increased metabolism and oxygen demand. This can result in elevated respiratory rate due to increased oxygen needs and tissue damage. Acidosis from lactic acid accumulation prompts faster breathing to remove excess acid. Lung damage or hypoxemia can occur, further increasing respiratory rate to compensate. Additionally, stress responses trigger hormonal release, boosting respiratory rate. Overall, the elevated respiratory rate in sepsis reflects efforts to combat infection, tissue repair, and oxygen demand, but it also indicates a serious condition requiring close clinical assessment, as lung damage and circulatory failure can impede oxygen exchange and utilization, leading to decreased oxygen saturation [40,41]. During sepsis, the metabolism of cells can increase, which can lead to acidosis. This can affect oxygen transport and lower oxygen saturation. Sepsis can often cause low blood pressure (hypotension). Low blood pressure can prevent enough blood from reaching the organs in the body, which can lower oxygen saturation. Therefore, it can be said that regular monitoring of oxygen saturation levels in a patient with sepsis and adjusting treatment, if necessary, will allow sepsis to be detected and prevented at an early stage.

Procalcitonin (PCT) is a host-directed biomarker used in the management of sepsis. PCT levels are used in many clinical situations, such as assessing the severity and extent of bacterial infections, differentiating sepsis from other inflammatory conditions, monitoring response to treatment, and managing antibiotic use. PCT levels can help in the early diagnosis of bacterial infections and in the differential diagnosis of sepsis from other inflammatory conditions. High PCT levels may indicate the presence of bacterial infections. Higher PCT levels can often indicate more serious bacterial infections. A further decline in PCT levels may indicate that the treatment is effective, and the infection is under control [42]. PCT levels can help manage the start and duration of antibiotic treatment. High PCT levels may indicate that bacterial infections persist, in which case antibiotic treatment can continue. However, low PCT levels may suggest that antibiotic treatment is unnecessary. PCT is considered a more specific marker, especially in bacterial infections. This may reduce the likelihood of PCT levels being confused with other conditions, such as viral infections [43,44]. Hence, PCT can be a timely, perfect, and effective diagnostic marker for sepsis brought on by bacterial infection [45].

There is a strong association between sepsis and positive blood cultures, and they play a critical role in the diagnosis of sepsis. Blood cultures are used to identify the causative microorganisms of an infection in the body. Positive blood cultures indicate that an infection in the body has spread into the bloodstream and bacteria have entered the blood. Positive blood cultures are also very useful in determining the severity of sepsis and how widespread the infection is. Different types and amounts of bacteria can affect the severity of sepsis. Positive blood cultures can also help to identify the source of focal infection [46,47,48].

Artificial intelligence studies, that contribute positively to the survival rates and treatment outcomes of patients by increasing the chances of early diagnosis and intervention in sepsis prediction, are frequently used in the literature [14,49,50]. However, in the current study, the performance criterion of the artificial intelligence model used to predict sepsis was significantly higher than the others. Therefore, XGBoost has a very high performance in predicting sepsis and the five proposed biomarkers will be very useful in the clinic for early diagnosis, treatment, and monitoring response to treatment.

## 5. Conclusions

The tree-based XGBoost algorithm proposed in this study can accurately distinguish and evaluate sepsis through selected biomarkers. A combination of XGBoost and XAI can provide a clear interpretation of the global risk estimate for sepsis and allow physicians to intuitively understand the impact of key biomarkers in the proposed model. As a result, research in which prediction models are used together with XAI is crucial, especially in medical applications, as it enhances the transparency and trustworthiness of the model predictions. It allows healthcare professionals to interpret and validate the model’s decisions, ultimately aiding in the decision-making process.

## Figures and Tables

**Table 1:** Descriptive statistics for the clinical biomarkers of sepsis.

Variable	Group				*p*-Value	ES
	Reference Values for No Sepsis	No Sepsis (*n* = 1012)	Reference Values for Sepsis	Sepsis		
				(*n* = 560)		
Age (years)		68 (25)		76.5 (18)	<0.001	0.0669 (Small)
Systolic blood pressure (mmhg)	120–180	136 (31)	<90 or >140	130 (36)	<0.001	0.0115 (Small)
Respiratory rate (breaths/min)	12–20	22 (6.915)	>20 or <12	25.35 (8)	<0.001	0.0677 (Small)
Oxygen saturation (%)	95–100	96 (3)	<92	94 (6)	<0.001	0.0709 (Small)
Heart rate (beats/min)	60–100	95 (24.625)	>100 or <60	100 (26)	<0.001	0.00991 (Small)
Body temperature (°C)	36.5–37.5	37.8 (1.4)	<36 or >38	38 (1.5)	0.01	0.00418 (Small)
Haemoglobin (g/L)	13.5–17.5 (Male)	132 (24.812)	<13.5	128 (24)	0.001	0.00701 (Small)
Leukocyte particle concentration (×10^9^ cells/L)	4–10	11.3 (6.3)	<4 or >12	13.1 (7.65)	<0.001	0.0307 (Small)
C-reactive protein(mg/L)	<5	91.5 (120.25)	>10	126 (148.25)	<0.001	0.0194 (Small)
Procalcitonin (ng/mL)	<0.5	0.13 (0.498)	>2.0	0.51 (3.88)	<0.001	0.0891 (Small)
Neutrophil–lymphocyte count ratio	<3.5	8 (9.45)	>10	13.013 (14.3)	<0.001	0.0755 (Small)
Lactate (mmol/L)	<2.0	1.6 (0.883)	>2.0	1.9 (1.253)	<0.001	0.039 (Small)

The values are reported by median (IQR); IQR: interquartile range; ES: effect size.

**Table 2:** The optimal hyper-parameters of models determined by Grid Search.

Model	Optimal Hyper-Parameters
LightGBM	n_estimators = 1000, learning_rate = 0.1, colsample_bytree = 0.8, subsample = 0.8
AdaBoost	n_estimators = 100, learning_rate = 0.1
XGBoost	n_estimators = 1000, learning_rate = 0.1, max_depth = 2, subsample = 0.8

**Table 3:** Results of ML models on original and SOMOTE-NC applied data in sepsis (Values in parentheses are 95% confidence interval (CI)).

Model/Metric		Accuracy	F1-Score	Sensitivity	Specificity	PPV	NPV	AUC	Brier Score
AdaBoost	Orijinal	0.732 (0.682–0.783)	0.813 (0.769–0.858)	0.92 (0.871–0.954)	0.407 (0.314–0.506)	0.729 (0.667–0.784)	0.746 (0.616–0.85)	0.782 (0.663–0.902)	0.121 (0.091–0.189)
	SMOTE-NC	0.869 (0.835–0.903)	0.871 (0.837–0.905)	0.882 (0.827–0.925)	0.856 (0.797–0.903)	0.859 (0.802–0.905)	0.879 (0.823–0.923)	0.917 (0.869–0.966)	0.027 (0.022–0.038)
LightGBM	Orijinal	0.746 (0.696–0.795)	0.824 (0.78–0.867)	0.936 (0.891–0.966)	0.417 (0.323–0.515)	0.735 (0.674–0.79)	0.789 (0.661–0.886)	0.799 (0.676–0.923)	0.104 (0.097–0.134)
	SMOTE-NC	0.888 (0.856–0.92)	0.89 (0.858–0.922)	0.895 (0.842–0.935)	0.88 (0.825–0.924)	0.885 (0.832–0.927)	0.89 (0.835–0.932)	0.931 (0.887–0.974)	0.025 (0.02–0.036)
XGBoost	Orijinal	0.766 (0.718–0.814)	0.834 (0.791–0.876)	0.925 (0.878–0.958)	0.491 (0.393–0.589)	0.759 (0.698–0.813)	0.791 (0.674–0.881)	0.815 (0.708–0.923)	0.080 (0.062–0.098)
	SMOTE-NC	0.898 (0.868–0.929)	0.901 (0.87–0.931)	0.905 (0.854–0.943)	0.891 (0.837–0.932)	0.896 (0.844–0.935)	0.901 (0.848–0.94)	0.94 (0.898–0.98)	0.018 (0.014–0.021)

AdaBoost: adaptive boosting; LightGBM: light gradient boosting; XGBoost: extreme gradient boosting; SMOTE-NC: Synthetic Minority Oversampling Technique for Nominal and Continuous; PPV: positive predictive value; NPV: negative predictive value; AUC: area under of the ROC curve; CI: confidence interval.

## Data Availability

In appropriate cases, it can be requested from the corresponding author.
